# The complete mitochondrial genome of *Orthaga achatina* (Lepidoptera: Pyralidae)

**DOI:** 10.1080/23802359.2021.1884016

**Published:** 2021-03-11

**Authors:** Bing Liu, Hui Sun, Qingbin Zhan, Yunpeng Gai

**Affiliations:** a Collaborative Innovation Center of Sustainable Forestry in Southern China, College of Forestry, Nanjing Forestry University, Nanjing, China; b Nanjing Forest Police College, Nanjing, China; c Key Lab of Molecular Biology of Crop Pathogens and Insects, Ministry of Agriculture, Institute of Biotechnology, Zhejiang University, Hangzhou, China

**Keywords:** *Orthaga achatina*, complete mitochondrial genome, phylogenomic tree, Lepidoptera, *Cinnamomum camphora*

## Abstract

*Orthaga achatina* Butler is an important pest of camphor trees in Asia. The complete mitochondrial genome of *O. achatina* was sequenced in this study, which was 15,150 bp in size and comprised of 13 protein-coding genes, 22 transfer RNA genes, 2 ribosomal RNA genes, and a control region. Besides, we used a phylogenomic approach to infer evolutionary relationships of *O. achatina* and 23 Lepidoptera species based on 13 conserved protein sequences of the mitochondrial genome. Our results underline the potential importance of mitochondrial genomes in comparative genomic analyses of Lepidoptera species and provide a robust evolutionary insight across the tree of Lepidoptera insects.

Pyralidae is the largest family in Lepidoptera, with more than 25,000 species in the world, some of which are pests of agricultural and forestry plants, such as *Orthaga* (Yang et al. 2020)*. Orthaga achatina* Butler (Lepidoptera: Pyralidae) is the most serious pest of camphor trees (*Cinnamomum camphora*) in China, Korea, Japan, and Malaysia (Wu 2006). *Orthaga achatina* can also feed on other Lauraceae plants, such as *Lindera glauca* and *Cinnamomum cassia*, causing serious defoliation (Long et al. 2017). Mitochondrial genome has high mutation rate and low DNA recombination rate, which is of great significance in insect phylogenetic analysis (Gai et al. 2020; Yang et al. 2020). However, the mitochondrial genome of *O. achatina* has not been publicly reported. Therefore, we used *de novo* sequencing technology to sequence the whole mitochondrial genome of *O. achatina* to understand its mitogenomic background and genetic evolution relationship.

In the present study, samples of *O. achatina* were collected from camphor trees (N31.16568^°^, E120.62638^°^) in Suzhou, Jiangsu Province, China in July 2020. Some of these samples were immediately frozen at −80 °C for sequencing analysis and others were preserved in the Entomological Lab of Nanjing Forestry University, and their specimen code is 2020NJEM1855-1860. The genomic DNA was extracted from *O. achatina* using CTAB (cetyltrimethylammonium Ammonium Bromide) method (Huanca-Mamani et al. 2015). The quality and quantity of DNA sample were evaluated using an Agilent Bioanalyzer (Agilent Technologies, CA, USA) and the Qubit 3.0 Fluorometer (Life Technologies, CA, USA). The sequencing libraries were sequenced on the Illumina HiSeq platform in a paired-end 2 × 150 bp mode (Illumina, San Diego, CA). The raw data was processed to remove adaptors and low-quality bases using Trimmomatic v0.36 with default parameters (Bolger et al. 2014). De novo assembly was performed by SPAdes version 3.14 with the parameter ‘-k 21,33,55,77,99,127 –careful’ (Bankevich et al. 2012). The complete mitochondrial genomes were annotated by MITOS WebServer (http://mitos.bioinf.uni-leipzig.de/index.py) (Bernt et al. 2013) and submitted to NCBI GenBank (GenBank accession number: MT916176).

The mitochondrial genome of *O. achatina* is 15,150 bp, with a nucleotide composition of 38. 9% A, 41.8% T, 11.4% C and 7.9% G. The mitochondrial genome of *O. achatina* comprised the entire set of 37 typical invertebrate mitochondrial genes consisting of 13 protein-coding genes (PCGs), 22 transfer RNA genes (tRNAs), 2 ribosomal RNA genes (rRNAs), and a control region (D-loop). The majority coding strand encoded 23 genes (9 PCGs and 14 tRNAs), whereas the minority coding strand encoded 14 genes (4 PCGs, 8 tRNAs, and 2 rRNAs). The sequence and arrangement of the genes are highly conserved, indicating that they are similar to the typical characteristics of Lepidoptera mitochondrial genome (Wu et al. 2016, 2020; Liu et al. 2018). A total of 44 overlapping nucleotides were found in 6 loci, ranging from 2 to 25 bp, and 857 bp intergenic nucleotides in 22 locations, ranging from 4 to 297 bp in length.

All protein-coding genes (PCGs) are initiated with ATN as the starting codon except the *cox1*, which is no justification for continued speculation about polynucleotide start codon similar to other Lepidoptera insects (Singh et al. 2017; Liu et al. 2018; Yang et al. 2020). Ten PCGs had canonical stop codons TAA or TAG, while three had incomplete termination codons single T (*cox3* and *atp6*) or TA (*nad4L*). There were 22 tRNA genes with a length between 63 and 70 bp. All tRNA genes exhibited a typical clover-leaf secondary structure, except for tRNA-Ser(AGN) lacking the dihydrouridine (DHU) arm, which is common in Lepidoptera insects (Garey and Wolstenholme 1989). The lengths of lrRNA and srRNA were 1362 bp and 780 bp, respectively. The control region was located between srRNA and tRNA-Met with a total length of 298 bp.

The mitochondrial genomes of 23 Lepidoptera species previously sequenced were downloaded from GenBank and compared with *O. achatina* in this study. To identify the orthogroups, the BLAST-based ortholog detector OrthoFinder v2.2.7 (Emms and Steven 2019) with default parameter values were used to identify ortholog among all the protein sequences of the 24 mitochondrial genomes. The phylogenetic relationship of *O. achatina* and 23 Lepidoptera species was inferred from phylogenetic analysis of the 13 protein coding genes using MEGA7.0 software with maximum likelihood method and 1000 replicate sets on bootstrap analysis. The amino acid identity (AAI) of the 13 protein coding genes of each Lepidoptera species and *O. achatina* were calculated by NCBI BLASTP. The phylogenetic tree and AAI heatmap of each protein was visualized using EVOLVIEW version 2 (https://evolgenius.info//evolview-v2) (He et al. 2016). Phylogenetic analyses showed similar relationships among sampled families as shown in Yang et al. (2020). Each clade showed a monophyletic cluster and the following clades were highly supported (Figure 1): (1) Pyralidae + Crambidae; and (2) (Pyralidae + Crambidae) + (Noctuidae + (Bombycidae + Geometridae)). We also found that *O. achatina* strains had the closest relationship with the genus *Hypsopygia* and *Endotricha*, which were located in a clade in the clade of Pyralidae. This study can provide a useful resource for the genetic evolution of *O. achatina* and underline the potential importance of mitochondrial genomes in comparative genomic analyses of Lepidoptera species.

**Figure 1.**
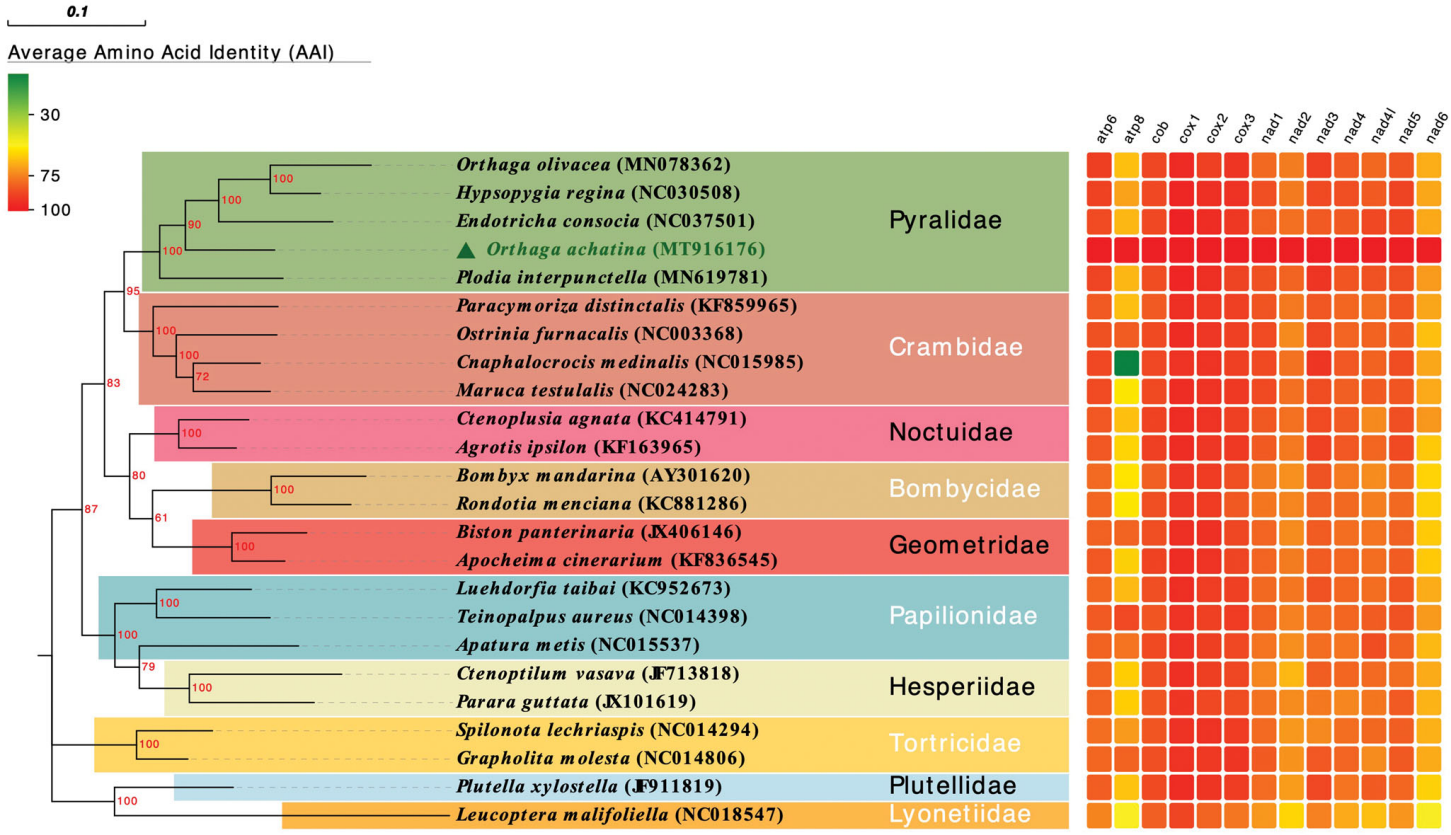
Phylogenetic relationship of the nine Lepidoptera families, which were inferred from phylogenetic analysis of the 13 proteins by using the maximum likelihood method of MEGA 7.0. The number above in each node indicates the bootstrap support values with 1000 replicates.

## Data Availability

The data that support the findings of this study are openly available in the National Center for Biotechnology Information under the BioProject accession number PRJNA669357 (https://www.ncbi.nlm.nih.gov/bioproject/669357). The figure is available on the figshare repository: https://doi.org/10.6084/m9.figshare.13154066. The complete mitochondrial genome and its annotation file are available in the Zenodo repository: http://doi.org/10.5281/zenodo.4159192.
